# Electrocardiographic and Echocardiographic Insights From a Prospective Registry of Asian Elite Athletes

**DOI:** 10.3389/fcvm.2021.799129

**Published:** 2022-01-03

**Authors:** Tee Joo Yeo, Mingchang Wang, Robert Grignani, James McKinney, Lay Pheng Koh, Frankie Hun Yau Tan, Gregory Chung Tsing Chan, Nigel Tay, Siew-Pang Chan, Chi-Hang Lee, David Oxborough, Aneil Malhotra, Sanjay Sharma, Arthur Mark Richards

**Affiliations:** ^1^Cardiac Department, National University Heart Centre Singapore, Singapore, Singapore; ^2^Cardiovascular Research Institute, National University Heart Centre Singapore, Singapore, Singapore; ^3^National University Hospital Sports Centre, National University Hospital, Singapore, Singapore; ^4^Department of Paediatrics, National University Hospital, Singapore, Singapore; ^5^SportsCardiologyBC, University of British Columbia, Vancouver, BC, Canada; ^6^Sport Science and Medicine Centre, Singapore Sport Institute, Sport Singapore, Singapore, Singapore; ^7^Department of Physiology, Yong Loo Lin School of Medicine, National University of Singapore, Singapore, Singapore; ^8^Family Medicine Department, Cavendish Doctors, Auckland, New Zealand; ^9^Sport and Exercise Sciences, Liverpool John Moores University, Liverpool, United Kingdom; ^10^Division of Cardiovascular Sciences, University of Manchester, Manchester NHS Foundation Trust, Manchester, United Kingdom; ^11^Manchester Institute of Health and Performance, Manchester, United Kingdom; ^12^Cardiology Clinical Academic Group, St George's University of London, London, United Kingdom

**Keywords:** athlete's heart, sports cardiology, registry, Asian athlete, cardiac remodeling

## Abstract

**Background:** Asian representation in sport is increasing, yet there remains a lack of reference values for the Asian athlete's heart. Consequently, current guidelines for cardiovascular screening recommend using Caucasian athletes' norms to evaluate Asian athletes. This study aims to outline electrocardiographic and echocardiographic characteristics of the Asian athlete's heart using a Singaporean prospective registry of Southeast (SE) Asian athletes.

**Methods and Results:** One hundred and fifty elite athletes, mean age of 26.1 ± 5.7 years (50% males, 88% Chinese), were evaluated using a questionnaire, 12-lead electrocardiogram (ECG) and transthoracic echocardiogram. All ECGs were analyzed using the 2017 International Recommendations. Echocardiographic data were presented by gender and sporting discipline. The prevalence of abnormal ECGs among SE Asian athletes was 6.7%—higher than reported figures for Caucasian athletes. The abnormal ECGs comprised mainly anterior T wave inversions (ATWI) beyond lead V2, predominantly in female athletes from mixed/endurance sport (9.3% prevalence amongst females). None had echocardiographic structural abnormalities. Male athletes had reduced global longitudinal strain compared to females (−18.7 ± 1.6 vs. −20.7 ± 2.1%, *p* < 0.001). Overall, SE Asian athletes had smaller left ventricular cavity sizes and wall thickness compared to non-Asian athletes.

**Conclusion:** SE Asian athletes have higher abnormal ECG rates compared to Caucasian athletes, and also demonstrate structural differences that should be accounted for when interpreting their echocardiograms compared to athletes of other ethnicities.

## Introduction

Asian athletes feature prominently in international competitive sport and continue to show improvement at the pinnacle of physical ability—the Olympic Games (1). Altogether, 48 countries combine to form Asia, the largest and most populous continent in the world with a total population exceeding 4.4 billion (2). Despite this enormous population base, there remains a dearth of data for the Asian athlete's ECG and echocardiogram.

The electrocardiogram (ECG) is instrumental in differentiating pathology from physiology in the athlete's heart (3). ECG screening in athletes over the past decade has seen the development of qualitative and quantitative cutoffs, as well as ethnicity-specific recommendations. For instance, anterior T wave inversions (ATWI) up to lead V2 are deemed physiological in Caucasian athletes, whereas in Black athletes, ATWI up to lead V4 are considered within normal limits when preceded by J-point elevation and convex ST segment elevation. These ECG recommendations have reduced false positive rates substantially while preserving sensitivity in identifying pathology in athletes (4). In parallel to the ECG, echocardiographic imaging of Caucasian and Black athletes has also progressed considerably, leading to the establishment of normal reference values (5).

Physiological ECG and echocardiographic limits for Asian athletes have yet to be defined, with current guidelines recommending using Caucasian athletes' norms to evaluate Asian athletes (6). This study aims to highlight ECG and echocardiographic characteristics for Asian athletes via a prospective registry of athletes from Southeast (SE) Asia. Singapore, an island city-state with a multi-ethnic SE Asian population of Chinese (74.1%), Malay (13.4%), Indian (9.2%), and others (3.3%), forms the base for this study (7).

## Methods

The Singapore Sports Cardiology Registry is a descriptive, cross-sectional, and prospective registry of active elite athletes aged ≥18 years representing the country in competitive sport regardless of sporting discipline. The study was carried out between January and October 2018 in the Singapore Sport Institute Sports Medicine Centre, which is the only ambulatory centre in the country that provides medical assessment and clearance for all national athletes prior to sport participation. One hundred and fifty consecutive elite athletes from 32 different sporting disciplines completed a questionnaire where data collected included: age, gender, ethnicity, sporting discipline, training history, medical history, and family history of sudden cardiac death.

### Electrocardiography

Standard resting 12-lead ECGs were performed on athletes in a supine position, using an ELI 230 ECG machine (Mortara, Milwaukee, WI) at a paper speed of 25 mm/s. All ECGs were analyzed in the digital unfiltered format for abnormalities based on established ECG interpretation criteria for athletes, namely the European Society of Cardiology recommendations in 2010 (ESC2010), the Seattle Criteria in 2013 (SC2013), the Refined Criteria in 2014 (RC2014) and the International recommendations in 2017 (IR2017) (8–11).

### Echocardiography

All recruited athletes underwent resting M-mode, two-dimensional (2D) and Doppler transthoracic echocardiography using a Vivid S6 ultrasound system (GE Healthcare, Milwaukee, WI), in accordance with American and British Society of Echocardiography guidelines (12–14). All echocardiograms were performed immediately after the resting 12-lead ECG. Left ventricular (LV) ejection fraction was measured using the biplane method of disks. Pulsed wave tissue doppler imaging of the septal, lateral LV and tricuspid annuli was performed in the apical 4-chamber view to obtain peak early (e′) and late (a′) myocardial velocities and the ratio of early diastolic transmitral flow velocity to e′ (E/e′).

Radiofrequency data from three cardiac cycles were stored and indices of myocardial deformation obtained by speckle-tracking analysis using EchoPac software (Version 11.1.8, GE Healthcare, Horten, Norway). Automated functional imaging was utilized to track and analyse peak systolic strain using a tri-plane imaging probe. Global longitudinal strain (averaged) was measured for all participants. Measures of right ventricular systolic function, 2D fractional area change and tricuspid annular plane systolic excursion (TAPSE), were also obtained.

LV geometry was classified into four groups based on American and European Society of Echocardiography guidelines: normal geometry, concentric remodeling, eccentric hypertrophy, and concentric hypertrophy (12, 14). This was based on cutoffs for relative wall thickness (abnormal > 0.42) and LV mass index (LV hypertrophy defined as LV mass index >95 g/m^2^ in females and >115 g/m^2^ in males).

All echocardiographic images were separately reviewed and analyzed by two independent cardiologists, blinded to the participants' ethnicity, sporting discipline, and training volume. Discrepancies in quantitative parameters were averaged and presented as mean values, whereas mutual discussion was carried out for qualitative differences.

### Statistical Analysis

The sample characteristics were presented as mean ± standard deviation (SD) or frequency (%), depending on their nature. The ECGs and echocardiographic data were presented by gender as well as sport discipline. Sport disciplines, based on the European Association of Preventive Cardiology (EAPC) position statement in 2017, were divided into two groups—skill and power (low to moderate oxygen consumption) vs. mixed and endurance (moderate to high oxygen consumption) (5). Independent *t*-tests were performed to ascertain if there were significant differences in ECGs and echocardiographic parameters between the groups. Regression analyses were carried out to examine the association of demographics (i.e., age, gender, ethnicity), sporting discipline and training hours on ECG abnormalities as well as commonly utilized echocardiographic parameters. Data analysis was performed with STATA version 14 (Statacorp, Texas) and all statistical tests were conducted with 5% level of significance.

Written informed consent was obtained from all participants. All study procedures conformed to the ethical guidelines of the 1975 Declaration of Helsinki.

## Results

### Baseline Characteristics

Of the 150 SE Asian athletes, there were 75 (50%) males, and the ethnic distribution was Chinese (88%), Malay (5.3%), Indian (3.3%), and others [Sikh, Indian-Chinese and Indonesian] (3.3%). These are reflective of ethnic groups found within SE Asia. Participants had a mean age of 26.1 ± 5.7 years. Their average training duration was 19.3 ± 8.8 h per week, with mean competitive experience of 8.5 ± 4.7 years (Table 1). Baseline demographics between Chinese and non-Chinese athletes were comparable.

**Table 1:** Baseline characteristics and sport classification of athletes.

	**Mean ± SD/Frequency (%)**
Age (years)	26.1 ± 5.7
Male gender	75 (50)
**Ethnic group**	
• Chinese	132 (88)
• Malay	8 (5.3)
• Indian	5 (3.3)
• Others	5 (3.3)
Body mass index (kg/m^2^)	22.9 ± 3.1
Body surface area (m^2^)	1.8 ± 0.2
Training hours/week	19.3 ± 8.8
Number of years competing	8.5 ± 4.7
**EAPC sport category**	
• Skill	20 (13.3)
• Power	24 (16)
• Mixed	50 (33.3)
• Endurance	56 (37.3)

### Electrocardiographic Data

The most common training-related changes based on IR2017 were: sinus bradycardia (65.3%), early repolarization (46%), and sinus arrhythmia (25.3%). Early repolarization (70.7 vs. 21.3%, *p* < 0.0001) and voltage criteria for LV hypertrophy (17.3 vs. 4%, *p* < 0.02) were more common in males than females. Athletes in mixed and endurance sports demonstrated more sinus bradycardia (73.6 vs. 45.4%, *p* = 0.001), incomplete right bundle branch block (15.1 vs. 2.3%, *p* = 0.024), early repolarization (53.8 vs. 27.3%, *p* = 0.004) and voltage criteria for LV hypertrophy (15.8 vs. 0%, *p* = 0.003) compared to those in skill and power-based sports. Borderline findings based on IR2017 were noted in 3 separate athletes −2 with left atrial enlargement and 1 with complete right bundle branch block.

Using ESC2010, 31 ECGs in our cohort were identified with abnormal findings unrelated to training (20.7% prevalence), and application of SC2013 reduced the number of abnormal ECGs to 13 (8.7% prevalence). Identical results for both RC2014 and IR2017 were obtained (6.7% prevalence). There were no differences in prevalence of abnormal ECGs between Chinese and non-Chinese athletes.

Characteristics of all 10 athletes with abnormal ECGs based on IR2017 are outlined in Table 2, and digital ECGs are available as Supplementary Material. All were Chinese and engaged in mixed or endurance sports. Of these, 8 athletes (7 females; 9.3% prevalence among female athletes) showed anterior T wave inversions (ATWI) in 2 contiguous leads beyond V1. Echocardiograms did not reveal any structural abnormalities in all 10 athletes apart from LV hypertrophy. Of 4 athletes who consented to undergo cardiac magnetic resonance imaging, no abnormalities were found.

**Table 2:** Characteristics of athletes with abnormal electrocardiograms based on the 2017 International Recommendations.

**No**	**Age (yrs)**	**Gender**	**Ethnicity**	**BSA (m^**2**^)**	**Sport**	**Weekly training hours**	**ECG abnormality**	**LVWT (mm)**	**LVIDD (mm)**	**LV mass (g)**	**LVEF (%)**	**GLS (%)**
1	23.4	Male	Chinese	1.87	Kayak	25	QRS 140 msec	12	55	288.9	58	−18
2^*^	26.4	Male	Chinese	1.80	Marathon	15	2 PVCs	12	60	280.7	56	−19
3	18.0	Female	Chinese	1.58	Tennis	27	ATWI V1-V3	9	44	148.7	58	−19
4^*^	36.2	Female	Chinese	1.44	Marathon	10	ATWI V1-V4	8	48	132.4	60	−20
5	26.1	Female	Chinese	1.82	Waterpolo	18	ATWI V1-V3	9	50	159.4	61	−22
6^*^	34.4	Female	Chinese	1.68	Netball	14	ATWI V1-V3	9	47	137.2	67	−23
7	32.2	Female	Chinese	1.72	Netball	10	ATWI V1-V3	7	47	108.8	53	−20
8	33.1	Female	Chinese	1.49	Marathon	10	ATWI V1-V3	7	48	111.3	63	−23
9^*^	38.8	Male	Chinese	1.47	Marathon	12	ATWI V1-V3	10	52	185.7	59	−20
10	26.7	Female	Chinese	1.52	Dragonboat	22	ATWI V1-V3	7	46	105.9	63	−23

*BSA, body surface area; ECG, electrocardiogram; LVWT, Left ventricular wall thickness; LVIDD, Left ventricular internal diameter in diastole; LVEF, Left ventricular ejection fraction; GLS, global longitudinal strain; PVC, premature ventricular contraction; ATWI, Anterior T wave inversion.*

* *Athletes with cardiac magnetic resonance imaging performed—no pathology detected*.

### Echocardiographic Data

Comprehensive structural and functional echocardiographic parameters for the cohort by gender and sport discipline are presented in Tables 3, 4, respectively. Normal geometry was encountered in the majority of athletes (77.3%, *n* = 116), followed by eccentric hypertrophy (*n* = 16, 10.7%), concentric remodeling (*n* = 13, 8.7%), and concentric hypertrophy (*n* = 5, 3.3%).

**Table 3:** Gender differences in echocardiographic parameters for Singapore athletes.

	**Male** (***n*** **= 75)**			**Female** (***n*** **= 75)**			***P***
	**Mean**	**SD**	**Min, Max**	**Mean**	**SD**	**Min, Max**	
**Left heart**							
LVWT (mm)	9.4	1.2	7, 13	7.7	1.1	5, 11	<0.001
LVWT/BSA (mm/m^2^)	5.1	0.7	3.6, 7.0	4.7	0.8	3.0, 7.2	0.002
LVIDD (mm)	51.6	3.7	43, 63	47.7	3.6	40, 57	<0.001
LVIDD/BSA (mm/m^2^)	27.6	2.4	23, 35	29.0	2.7	21, 34	0.001
LVPWD (mm)	9.6	1.3	6, 13	7.9	1.2	5, 11	<0.001
Relative wall thickness	0.37	0.05	0.24, 0.47	0.33	0.05	0.2, 0.5	<0.001
LV mass (g)	181.9	43.7	103, 316.2	122.1	29.3	64.5, 199.6	<0.001
LV mass/BSA (g/m^2^)	97.0	22.5	62, 160	74.2	17.7	39.3, 129.6	<0.001
LVEDV (ml)	127.4	22.1	77, 189	94.2	20.1	60, 154	<0.001
LVEDV/BSA (ml/m^2^)	68.1	11.7	97.7	56.9	10.9	83.2	<0.001
LV stroke volume (ml)	74.1	14.2	42, 117	56.2	12.4	33, 94	<0.001
LVEF (%)	58.2	4.1	47, 67	60.1	4.0	46, 67	0.004
LA (mm)	36.1	4.6	25, 48	33.0	3.7	25, 43	<0.001
LA/BSA (mm/m^2^)	19.3	2.6	14.6, 27.2	20.0	2.2	15.6, 26.5	NS
LA volume (ml)	65.4	19.0	36, 135	53.0	14.1	28, 96	<0.001
LA volume/BSA (ml/m^2^)	34.9	9.9	19.6, 70.6	32.1	7.8	17.2, 48.8	NS
E velocity (cm/s)	74.3	14.8	23, 106	84.6	15.1	55, 123	<0.001
DT (ms)	157.3	19.9	118, 208	149.8	19.4	106, 198	0.02
A velocity (cm/s)	39.8	8.0	21, 60	43.0	9.8	25, 69	0.03
E/A	1.9	0.5	0.5, 3.9	2.0	0.5	1.3, 4.2	NS
Septal annular E' (cm/s)	11.8	1.8	7, 16	11.9	1.8	8, 17	NS
Septal annular A' (cm/s)	7.2	1.5	4, 11	6.4	1.1	4, 9	0.001
Septal annular E'/A'	1.7	0.5	0.7, 3.5	1.9	0.4	1.1, 3	0.03
Septal annular E/E'	6.3	1.1	3.3, 8.8	7.3	1.5	4.1, 10.9	<0.001
Septal annular systolic velocity (cm/s)	8.4	1.0	6, 12	8.0	1.0	6, 11	0.01
Lateral annular E' (cm/s)	14.7	2.7	10, 23	15.0	2.5	9, 20	NS
Lateral annular A' (cm/s)	6.8	1.4	4, 11	6.6	1.2	4, 9	NS
Lateral annular E'/A'	2.3	0.7	1.1, 3.8	2.3	0.6	1.3, 4	NS
Lateral annular E/E'	5.1	1.0	2.3, 7.8	5.8	1.2	3.3, 9.8	<0.001
Lateral annular systolic velocity (cm/s)	10.3	2.0	6, 15	10.0	1.6	7, 14	NS
GLS (%)	−18.7	1.6	−16, −22	−20.7	2.1	−15, −24	<0.001
SoV diameter (mm)	32.6	3.5	26, 40	27.5	2.5	22, 35	<0.001
SoV diameter/BSA (mm/m^2^)	17.4	1.9	13.7, 21.4	16.7	1.8	12.6, 21.6	0.02
**Right heart**							
RA volume (ml)	64.6	23.4	25, 155	44.1	13.9	25, 95	<0.001
RA volume/BSA (ml/m^2^)	34.4	12.3	14.1, 84	26.6	7.8	15.2, 52.4	<0.001
RVOTpl (mm)	29.6	3.7	22, 40	27.2	4.0	16, 36	<0.001
RVOTp (mm)	33.5	4.7	22, 46	30.5	5.2	20, 49	<0.001
RVOTd (mm)	23.8	2.9	15, 30	21.9	2.9	14, 30	<0.001
RVD basal (mm)	39.2	5.4	14, 54	35.1	3.8	26, 44	<0.001
RVD mid (mm)	34.4	4.2	26, 50	30.6	4.2	20, 42	<0.001
RVD longitudinal (mm)	80.6	7.2	64, 97	71.6	8.0	51, 91	<0.001
TAPSE (mm)	25.1	3.4	19, 37	24.4	3.0	18, 32	NS
RV E' (cm/s)	12.6	2.3	7, 18	12.9	2.0	9, 19	NS
RV A' (cm/s)	8.8	2.3	3, 16	8.6	2.4	5, 15	NS
RV E'/A'	1.5	0.5	0.8, 3.7	1.6	0.5	0.7, 3.2	NS
RV sys vel (cm/s)	12.2	1.8	8, 19	11.6	1.4	9, 15	0.02
Normal	54	(72)			62	(82.7)	NS
Concentric remodeling	10	(13.3)			3	(4)	
Eccentric hypertrophy	8	(10.7)			8	(10.7)	
Concentric hypertrophy	3	(4)			2	(2.7)	

*LVWT, left ventricular wall thickness; LVIDD, left ventricular internal diameter in diastole; BSA, body surface area; PWD, posterior wall thickness in diastole; EDV, end diastolic volume; EF, ejection fraction; CO, cardiac output; CI, cardiac index; LA, left atrium; RA, right atrium; RVOT, right ventricular outflow tract; pl, parasternal long axis; p, proximal; d, distal; RVD, right ventricular diameter; DT, deceleration time; TAPSE, tricuspid annular plane systolic excursion; GLS, global longitudinal strain; SoV, Sinus of Valsalva*.

**Table 4:** Differences in echocardiographic parameters of Singapore athletes based on sporting discipline.

	**Skill and power**		**Mixed and endurance**		***P value***
	**(*n* = 44)**		**(*n* = 106)**		
	**Mean**	***SD***	**Mean**	***SD***	
**Left heart**					
LVWT (mm)	7.8	1.3	8.9	1.4	<0.001
LVWT/BSA (mm/m^2^)	4.5	0.6	5.0	0.8	<0.001
LVIDD (mm)	48.1	4.1	50.3	4.0	0.004
LVIDD/BSA (mm/m^2^)	27.6	2.4	28.6	2.7	0.025
LVPWD (mm)	8.0	1.3	9.1	1.4	<0.001
Relative wall thickness	0.33	0.05	0.36	0.05	0.002
LV mass (g)	127.3	39.8	162.3	47.1	<0.001
LV mass/BSA (g/m^2^)	71.9	17.3	91.3	23.0	<0.001
LVEDV (ml)	98.2	24.3	116.0	26.2	<0.001
LVEDV/BSA (ml/m^2^)	55.2	8.8	65.5	12.7	<0.001
LV stroke volume (ml)	58.0	13.7	68.2	16.1	<0.001
LVEF (%)	59.5	4.0	59.0	4.2	NS
LA (mm)	32.3	4.3	35.5	4.2	<0.001
LA/BSA (mm/m^2^)	18.5	1.7	20.2	2.4	<0.001
LA volume (ml)	48.3	14.3	63.7	17.2	<0.001
LA volume/BSA (ml/m^2^)	27.3	6.3	36.0	8.7	<0.001
E velocity (cm/s)	81.3	18.3	78.7	14.6	NS
DT (ms)	153.9	19.5	153.4	20.2	NS
A velocity (cm/s)	42.7	9.2	40.9	8.9	NS
E/A	2.0	0.6	2.0	0.5	NS
Septal annular E' (cm/s)	12.1	2.0	11.7	1.7	NS
Septal annular A' (cm/s)	7.1	1.5	6.7	1.4	NS
Septal annular E'/A'	1.8	0.5	1.8	0.5	NS
Septal annular E/E'	6.7	1.5	6.8	1.4	NS
Septal annular systolic velocity (cm/s)	8.2	1.0	8.3	1.0	NS
Lateral annular E' (cm/s)	15.1	2.5	14.7	2.6	NS
Lateral annular A' (cm/s)	6.6	1.4	6.7	1.3	NS
Lateral annular E'/A'	2.4	0.6	2.3	0.6	NS
Lateral annular E/E'	5.4	1.4	5.4	1.1	NS
Lateral annular systolic velocity (cm/s)	10.6	1.7	9.9	1.8	0.038
GLS (%)	−20.0	2.2	−19.6	2.0	NS
SoV diameter (mm)	29	3.8	30.5	3.9	NS
SoV diameter/BSA (mm/m^2^)	16.6	1.6	17.3	1.9	NS
**Right heart**					
RA volume (ml)	42.9	15.7	59.1	22.3	<0.001
RA volume/BSA (ml/m^2^)	24.0	6.5	33.2	11.3	<0.001
RVOTpl (mm)	26.5	4.4	29.2	3.6	0.001
RVOTp (mm)	30.3	6.2	32.7	4.6	0.024
RVOTd (mm)	21.7	3.0	23.3	3.0	0.006
RVD basal (mm)	35.1	4.2	37.9	5.2	0.001
RVD mid (mm)	31.1	4.0	33.1	4.7	0.01
RVD longitudinal (mm)	74.3	9.4	76.9	8.5	NS
TAPSE (mm)	23.9	2.9	25.1	3.3	0.033
RV E' (cm/s)	13.0	2.1	12.6	2.2	NS
RV A' (cm/s)	8.3	2.1	8.9	2.4	NS
RV E'/A'	1.6	0.5	1.5	0.5	NS
RV sys vel (cm/s)	11.7	1.6	12.0	1.7	NS

*LVWT, left ventricular wall thickness; LVIDD, left ventricular internal diameter in diastole; BSA, body surface area; PWD, posterior wall thickness in diastole; EDV, end diastolic volume; EF, ejection fraction; CO, cardiac output; CI, cardiac index; LA, left atrium; RA, right atrium; RVOT, right ventricular outflow tract; pl, parasternal long axis; p, proximal; d, distal; RVD, right ventricular diameter; DT, deceleration time; TAPSE, tricuspid annular plane systolic excursion; GLS, global longitudinal strain; SoV, Sinus of Valsalva*.

Of the entire cohort, 3 athletes (2% prevalence) were found to have minor structural heart disease that did not impact sporting participation: secundum atrial septal defect (male kayaker), dilated aortic root (male cyclist), and anterior mitral valve leaflet prolapse with mild mitral regurgitation (female triathlete).

### Differences Between Genders

**Figure 1:**
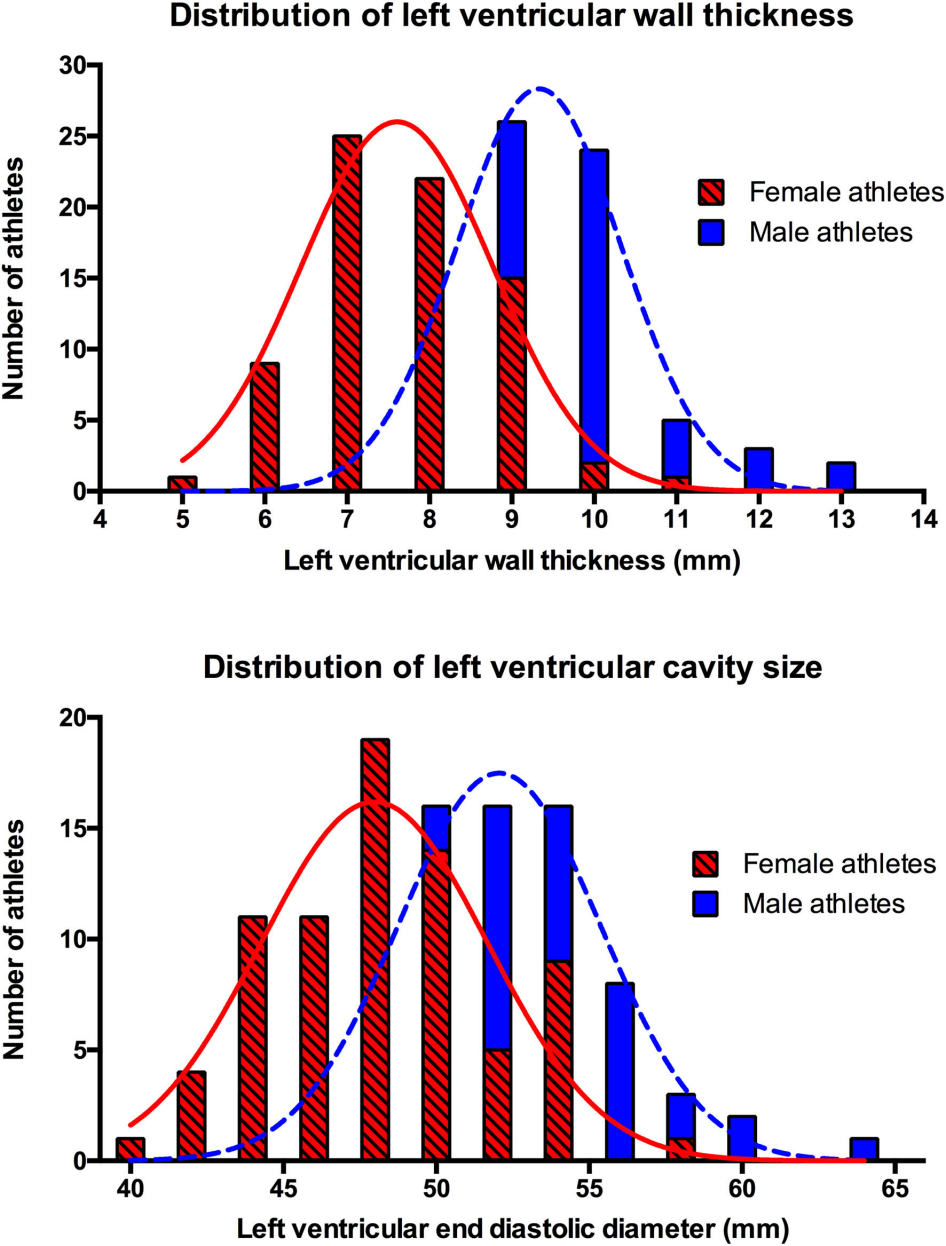
Distribution of left ventricular wall thickness and left ventricular cavity size by gender.

Absolute LV wall thickness (LVWT), LVIDD, volumes and mass were significantly larger in male compared to female athletes (Table 3 and Figure 1). In male athletes, diastolic indices, and global longitudinal strain were of smaller magnitudes compared to female athletes. There were no differences between male and female athletes in terms of LV geometry.

### Differences Between Sport Disciplines

Athletes from mixed or endurance sports demonstrated significantly increased left and right heart dimensions (i.e., LVWT, LVIDD, LV mass, LV end diastolic volume, LV stroke volume, left and right atrial volumes) compared to those from skill or power-based sports. Diastolic function and global longitudinal strain did not differ significantly between the two groups (Table 4).

### Differences Between Ethnic Groups

Echocardiographic parameters in Chinese and non-Chinese athletes were comparable for almost all variables.

### Regression Analyses

Logistic regression analysis showed that increasing age was an independent predictor for ATWI beyond V2 (odds ratio 1.13, *p* = 0.03) after adjusting for baseline demographics (age, gender, ethnicity, type of sport, training hours). Linear regression analysis showed that male gender and mixed and endurance sport disciplines were independently associated with a statistically significantly higher LVIDD, LVWT, and LV end diastolic volume after adjusting for baseline demographics.

## Discussion

This prospective electrocardiographic and echocardiographic registry is the first to evaluate a well-defined cohort of SE Asian elite athletes. It utilizes the contemporary IR2017 and describes a comprehensive range of ECG and echocardiographic characteristics, including values indexed to body surface area. Notably, the prevalence of ATWI beyond lead V2 on ECG was noted to be high at 9.3% for female athletes.

### Electrocardiographic Parameters

#### Comparison With Other Athletic Cohorts

**Figure 2:**
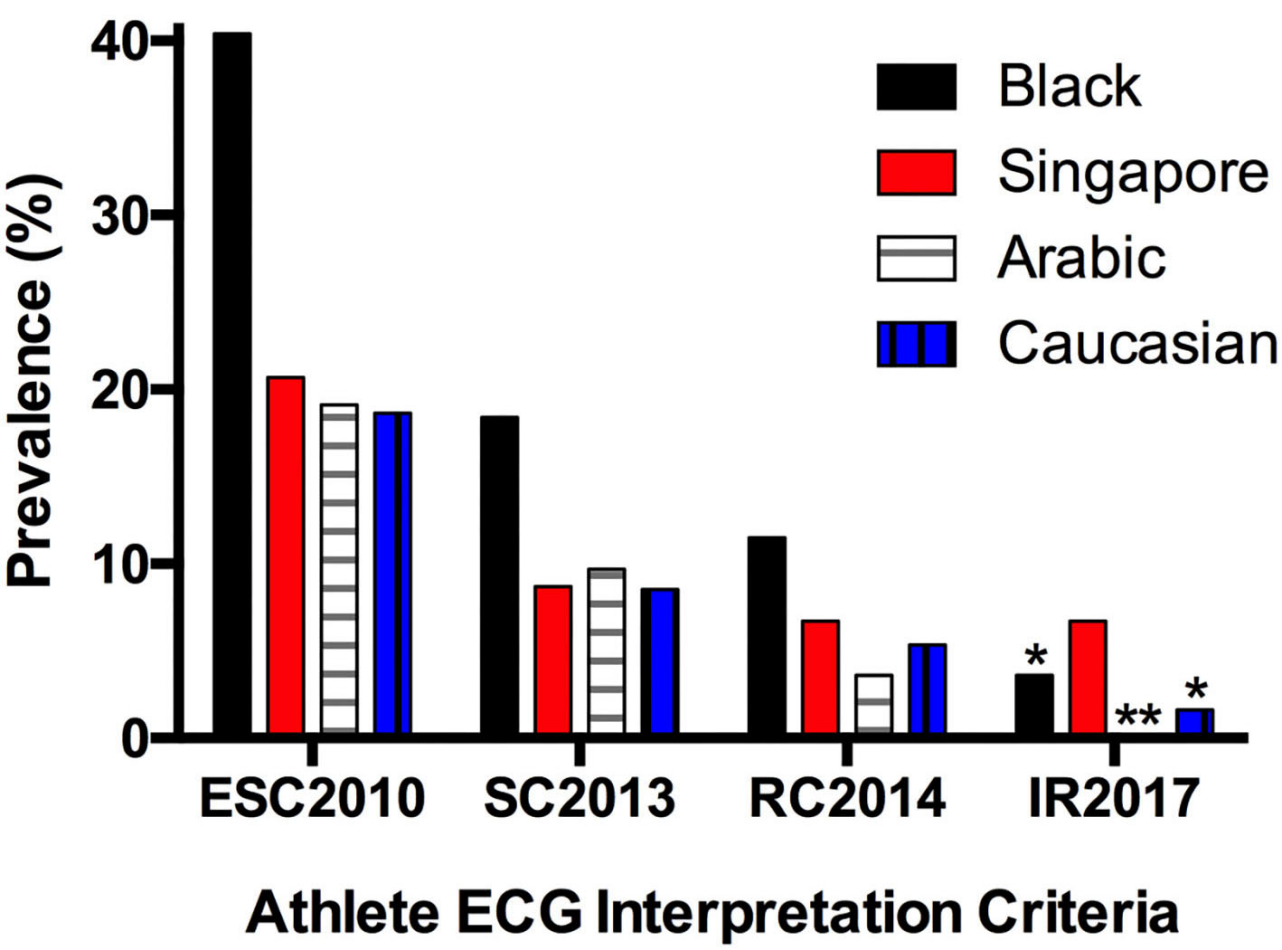
Prevalence of abnormal electrocardiograms in athletes of different ethnicities (10, 15, 16) (*Adolescent athletes; **Data unavailable; ESC, European Society of Cardiology; SC, Seattle Criteria; RC, Refined Criteria; IR, International Recommendations).

In non-Asian athletes, evolution of ECG interpretation criteria has led to considerable reduction in prevalence of abnormal ECGs while preserving sensitivity for detection of pathology. Prior to the current gold standard IR2017, its predecessor the RC2014 was used to compare athletes from different ethnicities. Reported prevalence rates of abnormal ECGs in Black, Caucasian and Arabic athletes using RC2014 (11.5, 5.3, and 3.6%, respectively) were substantially lower than those with ESC2010 and SC2013 (10, 15). Likewise, our cohort mirrors this trend of reduction in abnormal ECG with application of each newer criterion (Figure 2).

Application of IR2017 in a cohort of more than 11,000 adolescent soccer players revealed prevalence rates of abnormal ECGs at 3.6 and 1.6% for black and white athletes, respectively (16). Comparatively, the prevalence of abnormal ECGs in our cohort did not reduce further from RC2014 to IR2017 largely because our population was older in age and unaffected by the inclusion of the juvenile ECG pattern in IR2017 (17).

At 6.7%, the prevalence of abnormal ECGs in our SE Asian athletes is higher than reported figures for most non-Asian athletic populations. This is accounted for predominantly by ATWI beyond lead V2. Specifically, ATWI beyond V2 amongst SE Asian female athletes has a prevalence of 9.3%, more than 4 times the corresponding prevalence of 2.1% in a large cohort of Caucasian female athletes from UK (18).

Whereas, ATWI in non-Asian athletes may prompt further evaluation for possible cardiomyopathy (e.g., hypertrophic cardiomyopathy or arrhythmogenic cardiomyopathy), the incidence of pathological conditions manifesting with ATWI is very low in Singapore (19). For instance, the prevalence of hypertrophic cardiomyopathy in a large unselected young Singapore male population was 0.005% (20). In addition, incidence of sports-related sudden death was <1 in 2 million (21). These data may suggest a physiological rather than pathological explanation for the high prevalence of ATWI. A possible hypothesis is the displacement of the cardiac apex following longstanding endurance activity, which may be further influenced by unique contributions from gender and ethnicity (22).

The combined impact of ethnicity, gender, and sport discipline on ATWI deserves validation in a larger cohort. Confirmation of the high prevalence of ATWI up to lead V3 in Asian female endurance athletes combined with normal cardiac function and structure may lead to further refinement of ECG interpretation criteria with additional ethnic-specific cutoffs.

### Echocardiographic Parameters

Our study identified gender differences between athletes in global longitudinal strain, where male athletes have reduced GLS compared to females (an absolute difference of 2%). These gender differences corroborate findings by Park et al. in their study of an international cohort of university athletes and provide further insights into gender disparities in physiological cardiac remodeling (23).

#### Comparison With Other Athletic Cohorts

**Figure 3:**
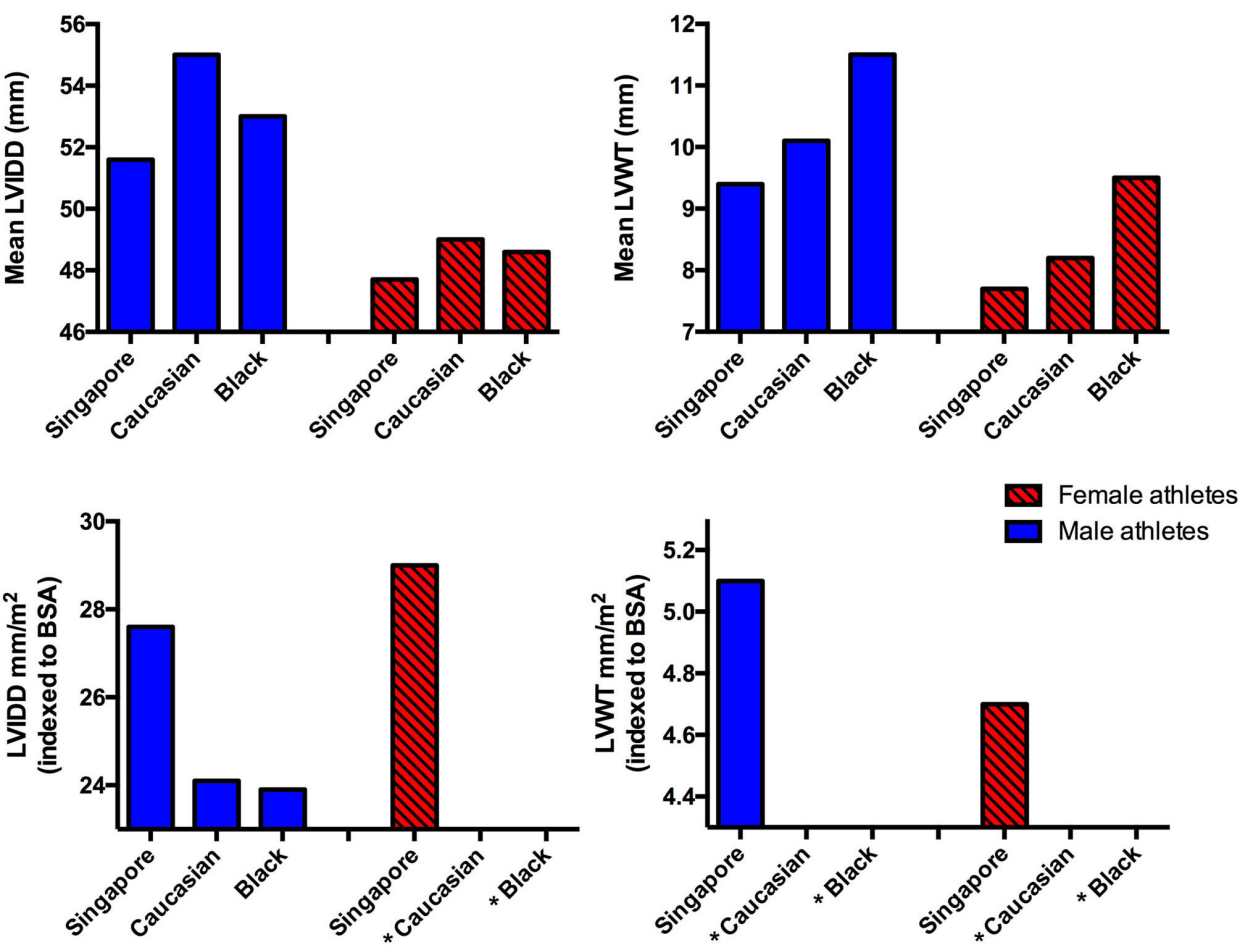
Comparison of mean and indexed left ventricular cavity size and left ventricular wall thickness in athletes from different ethnic groups (5, 6, 24–26) (*Data unavailable).

Compared to Caucasian and Black athletes, both male and female SE Asian athletes had smaller absolute LVIDD and LVWT (5, 6, 24–26) (Figure 3). A likely explanation for this is the proportionately smaller body sizes of SE Asians in general. However, the opposite was true when indexed values were used. Amongst male athletes, indexing of LVIDD to body surface area revealed that a cohort of Caucasian and Black professional basketball players from the United States had smaller dimensions compared to our cohort (25). This may be due to the majority (more than 40%) of our cohort consisting of endurance athletes. Athletes from endurance sports typically develop larger LVIDD compared to strength-trained athletes or those engaged in mixed sport such as basketball (27, 28).

Our SE Asian athlete cohort LVIDD and LVWT values corroborate with a group of Singapore athletes studied in 2004 and a group of mixed Asian university athletes in 2015 (29, 30). However, when compared to athletes from China and Japan, varying limits were observed with no discernable pattern identified. Male athletes from China and Japan had larger absolute LVIDD than those from our cohort. Nonetheless, this pattern was no longer present after indexing to body surface area (31, 32). China athletes also had higher absolute LVWT compared to our cohort, although the difference between cohorts was reversed after indexing for body surface area.

These differences between varying Asian cohorts underscore the impact of ethnicity on cardiac remodeling in athletes. Riding et al. demonstrated substantial variability in electrical and structural remodeling amongst Black athletes from different parts of the world, highlighting the influence of geographic origin (33). Their findings mirror our comparisons between SE Asian athletes and other East Asian cohorts. This suggests that, despite sharing a common ancestry, Asian athletes from different geographic locations have intrinsic differences in physiological electrical and structural cardiac remodeling. These differences warrant additional study in other Asian cohorts such as Malay, Thai, Filipino and Indonesian athletes, to avoid generalization of findings for all athletes of Asian descent.

Moreover, comparison of echocardiographic parameters indexed to body surface area is challenging as these are uncommonly reported in existing athlete cohorts. We encourage researchers involved in athlete registries to include indexed values to facilitate comparison of echocardiographic parameters.

### Limitations

Our registry of Singapore athletes has a sample size of 150 with predominant Chinese ethnicity, limiting applicability to all Asian athletes. Although non-Chinese athletes make up 12% of the cohort, these ethnic groups (Malay, Indian, Sikh, Indonesian) reflect the multi-racial population in Singapore and SE Asia. Importantly, comparison between Chinese and non-Chinese athletes did not reveal any clinically significant differences in demographics, prevalence of abnormal ECG or echocardiographic parameters. Within our cohort, representation from athletes engaging in power-based sports is also limited, with power-trained athletes making up 16% of the cohort.

Not all athletes with abnormal ECGs received comprehensive evaluation beyond echocardiography, for instance cardiac magnetic resonance imaging, exercise testing or 24 h electrocardiographic monitoring, to definitively exclude cardiac pathology. However, none of the athletes with abnormal ECGs were noted to have abnormal symptoms, examination findings or unexplained drop in fitness during their routine 2-yearly pre-competition clinical evaluations to date.

Given the small number of athletes with ATWI, regression analyses should be interpreted with caution.

Finally, we did not perform comparison with matched Caucasian and/or Black athletes to highlight ethnic-specific differences in ECG and echocardiographic characteristics.

## Conclusion

This prospective sports cardiology registry highlights electrocardiographic and echocardiographic characteristics in a cohort of SE Asian elite athletes as well as differences between other Asian and non-Asian cohorts. Further large-scale prospective registries are necessary to identify aspects of cardiac remodeling unique to athletes of different ethnic backgrounds.

## Data Availability Statement

The original contributions presented in the study are included in the article/Supplementary Materials, further inquiries can be directed to the corresponding authors.

## Ethics Statement

This study was reviewed and approved by National Healthcare Group Domain Specific Review Board Singapore (2017/00319) and Singapore Sports Institute Institutional Review Board (PH/EXP/018). All participants provided their written informed consent to participate in this study.

## Author Contributions

LK performed the measurements. MW, RG, FT, and GC were involved in planning and recruitment of participants. TY processed the experimental data, performed the analysis, drafted the manuscript, and designed the figures. NT edited the manuscript. S-PC aided in statistical analysis and interpreting the results. JM, C-HL, DO, AM, SS, and AR provided critical feedback and helped shape the research, analysis, and manuscript. All authors discussed the results and contributed to the final manuscript.

## Funding

This study was supported by a National University Health System Clinician Scientist Program award (N-171-000-469-001), and a centre grant (CGAug16M008) from the National Medical Research Council.

## Conflict of Interest

The authors declare that the research was conducted in the absence of any commercial or financial relationships that could be construed as a potential conflict of interest.

## Publisher's Note

All claims expressed in this article are solely those of the authors and do not necessarily represent those of their affiliated organizations, or those of the publisher, the editors and the reviewers. Any product that may be evaluated in this article, or claim that may be made by its manufacturer, is not guaranteed or endorsed by the publisher.
