# Supplementary material for: Electrocardiographic and Echocardiographic Insights From a Prospective Registry of Asian Elite Athletes
Source: Front Cardiovasc Med. 2022 Jan 3;8:799129. doi: 10.3389/fcvm.2021.799129 (PMC8761771; doi:10.3389/fcvm.2021.799129)
Supplement: Supplementary file 1 [file Data_Sheet_1.PDF]

,
ID:
DOB: 13-Sep-1994
23yr, Male

Vent rate: 53 BPM
PR int: 170 ms
QRS dur: 140 ms
QT/QTc: 435 / 419 ms
P-R-T axes: 68 71 1

Reviewed by ________________

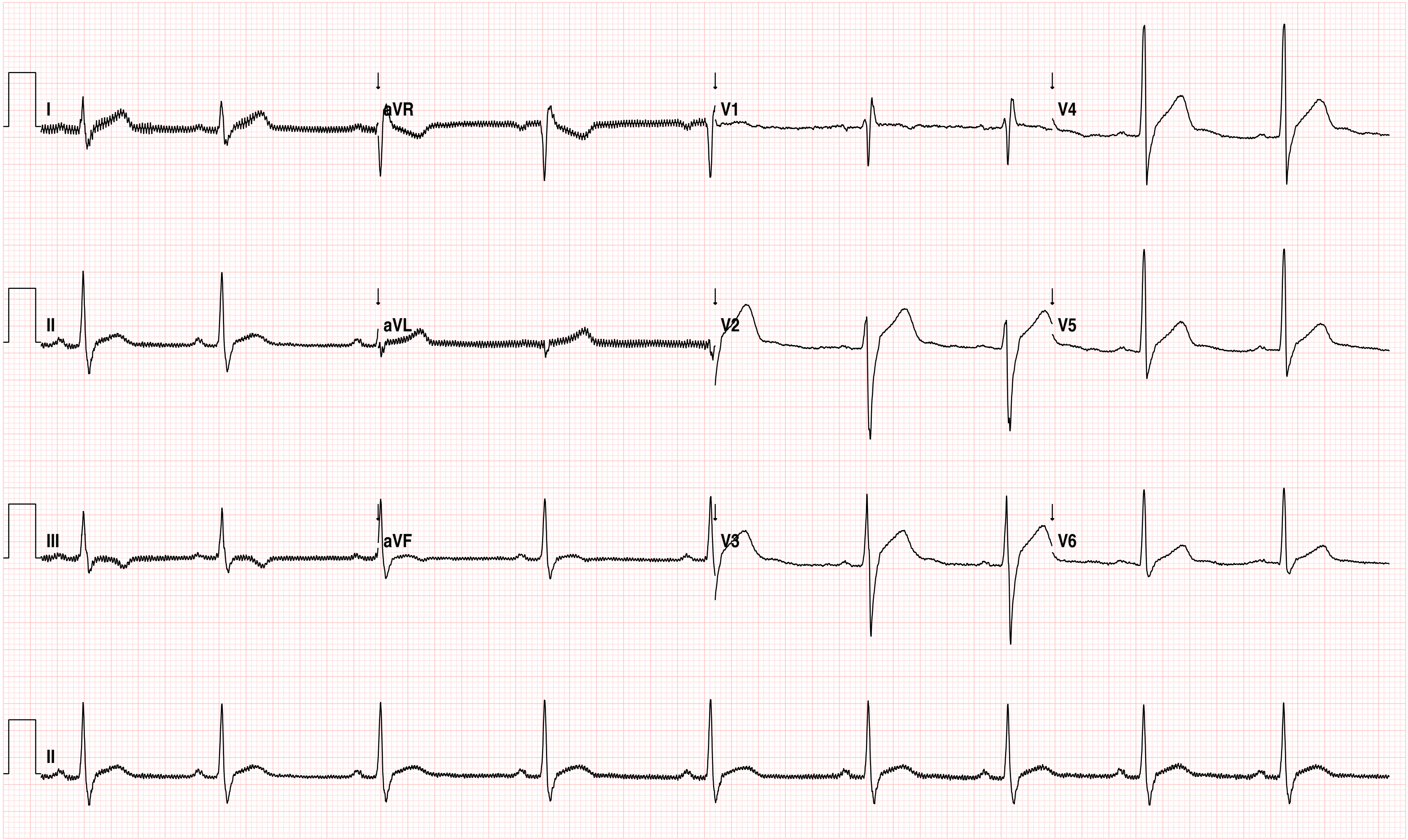

Site # 0 Cart # 0 ELI Link 4.11.6.0 Sequence # 00016 25mm/s 10mm/mV 0.05-300 Hz W

,
ID:
DOB: 06-Aug-1991
26yr, Male

Vent rate: 70 BPM
PR int: 162 ms
QRS dur: 97 ms
QT/QTc: 466 / 488 ms
P-R-T axes: 78 49 15

Reviewed by _______________

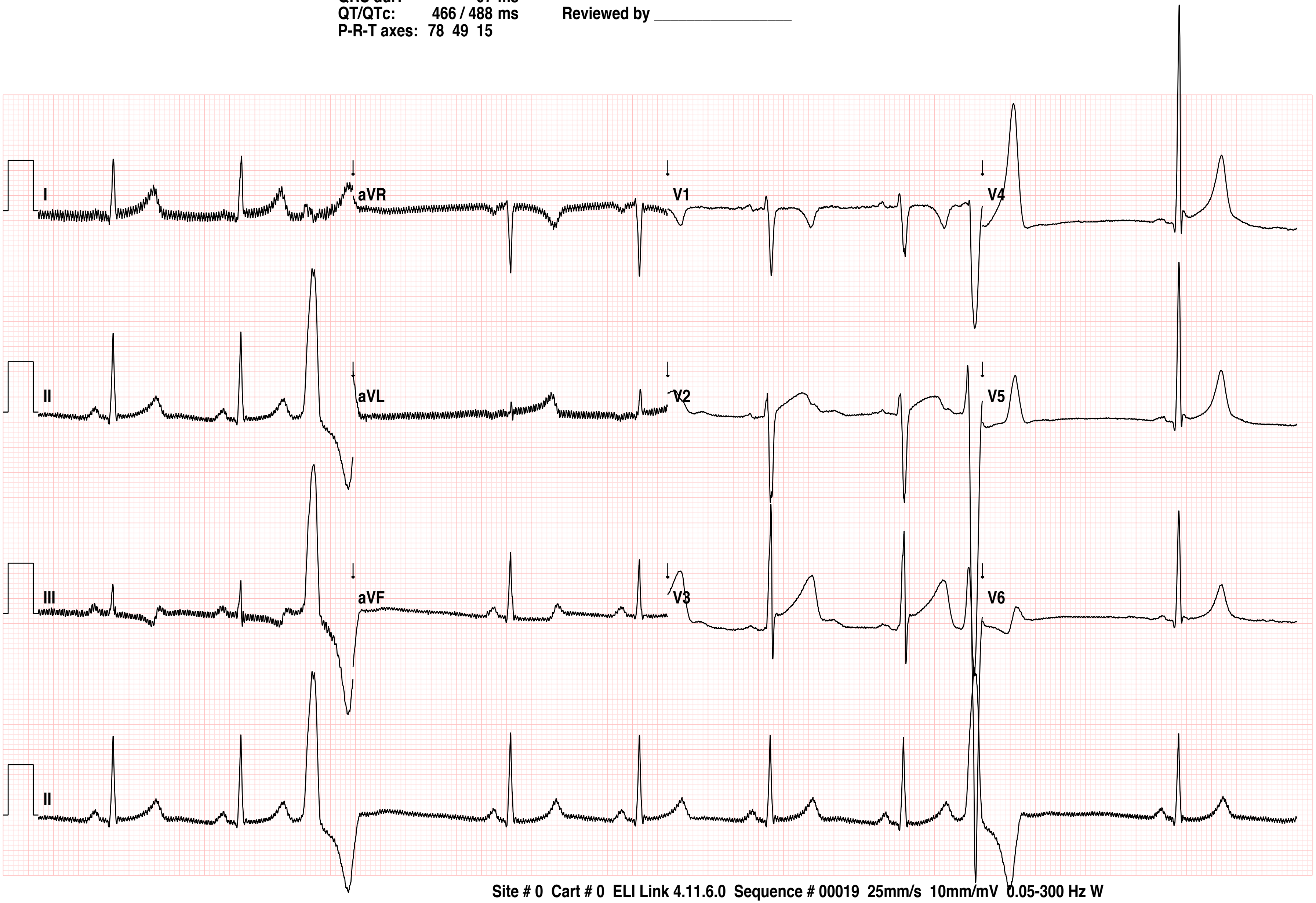

Site # 0 Cart # 0 ELI Link 4.11.6.0 Sequence # 00019 25mm/s 10mm/mV 0.05-300 Hz W

,
ID:
DOB: 25-Feb-2000
18yr, Female

Vent rate: 60 BPM
PR int: 160 ms
QRS dur: 94 ms
QT/QTc: 416 / 418 ms
P-R-T axes: 3 79 38

Reviewed by ________________

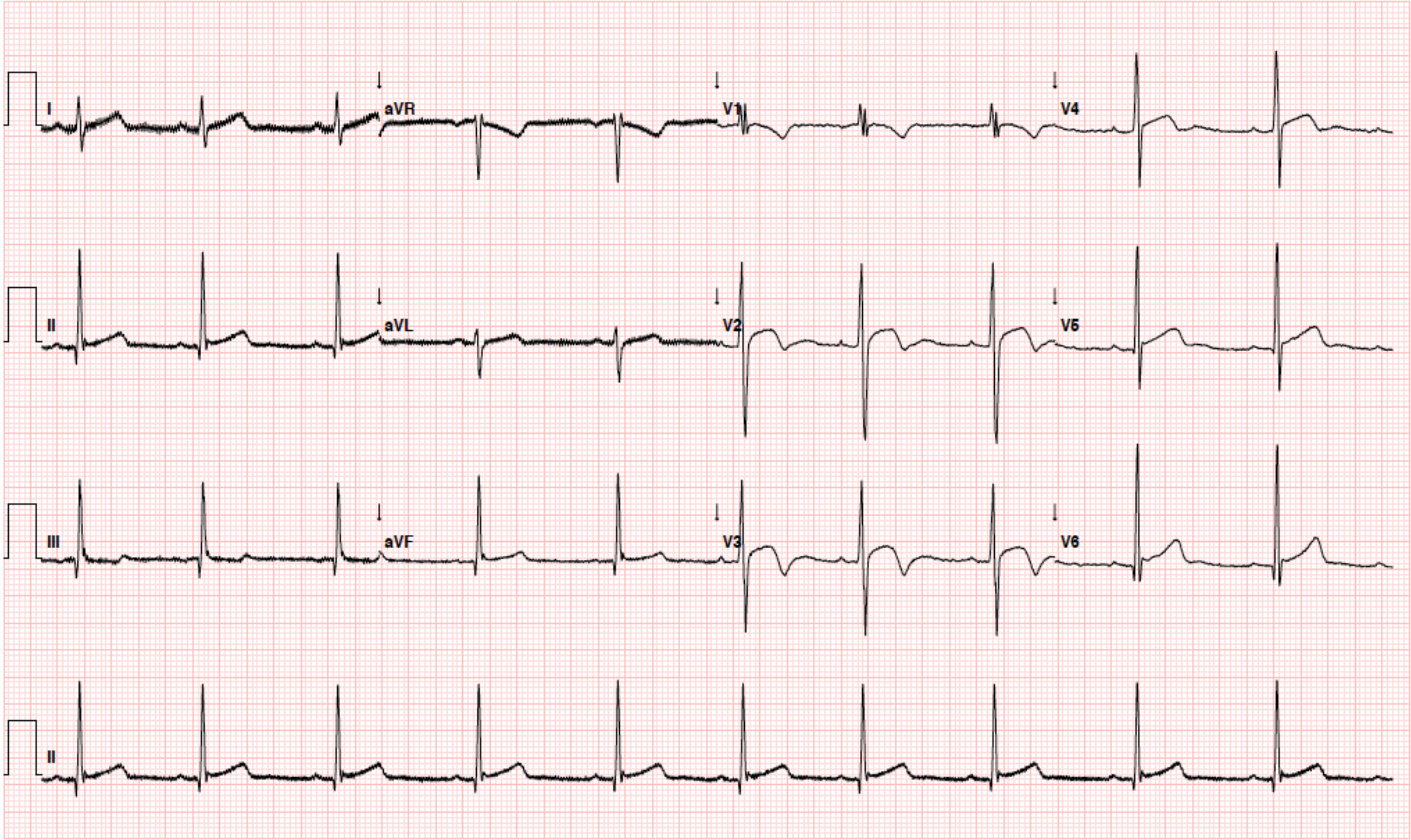

Site # 0 Cart # 0 ELI Link 4.11.6.0 Sequence # 00060 25mm/s 10mm/mV 0.05-300 Hz W

,
ID:
DOB: 31-Jan-1982
36yr, Female

Vent rate: 43 BPM
PR int: 128 ms
QRS dur: 105 ms
QT/QTc: 487 / 434 ms
P-R-T axes: 55 54 7

Reviewed by ________________

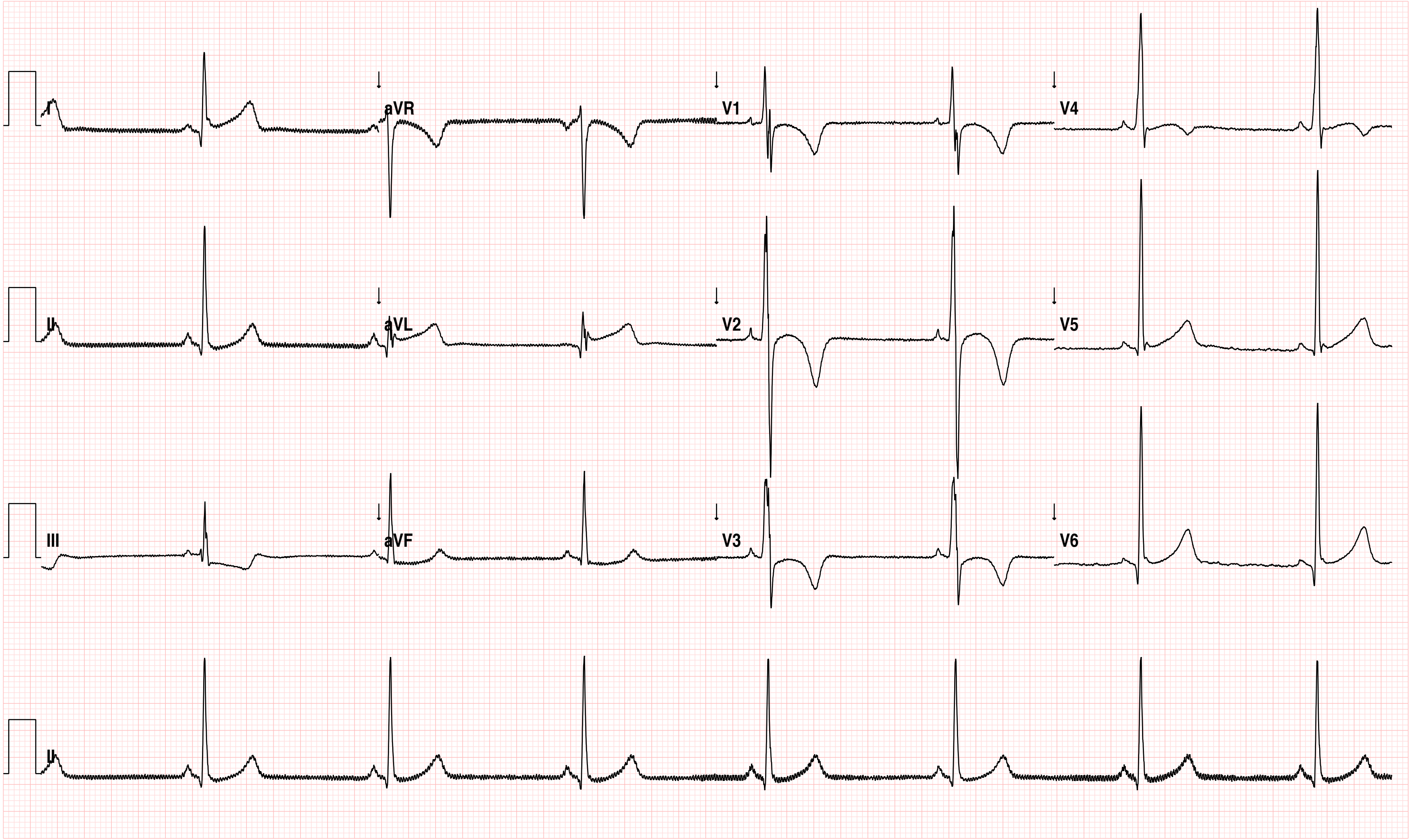

Site # 0 Cart # 0 ELI Link 4.11.6.0 Sequence # 00100 25mm/s 10mm/mV 0.05-300 Hz W

,
ID:
DOB: 09-Mar-1992
26yr, Female

Vent rate: 66 BPM
PR int: 151 ms
QRS dur: 83 ms
QT/QTc: 448 / 462 ms
P-R-T axes: 44 96 56

Reviewed by ________________

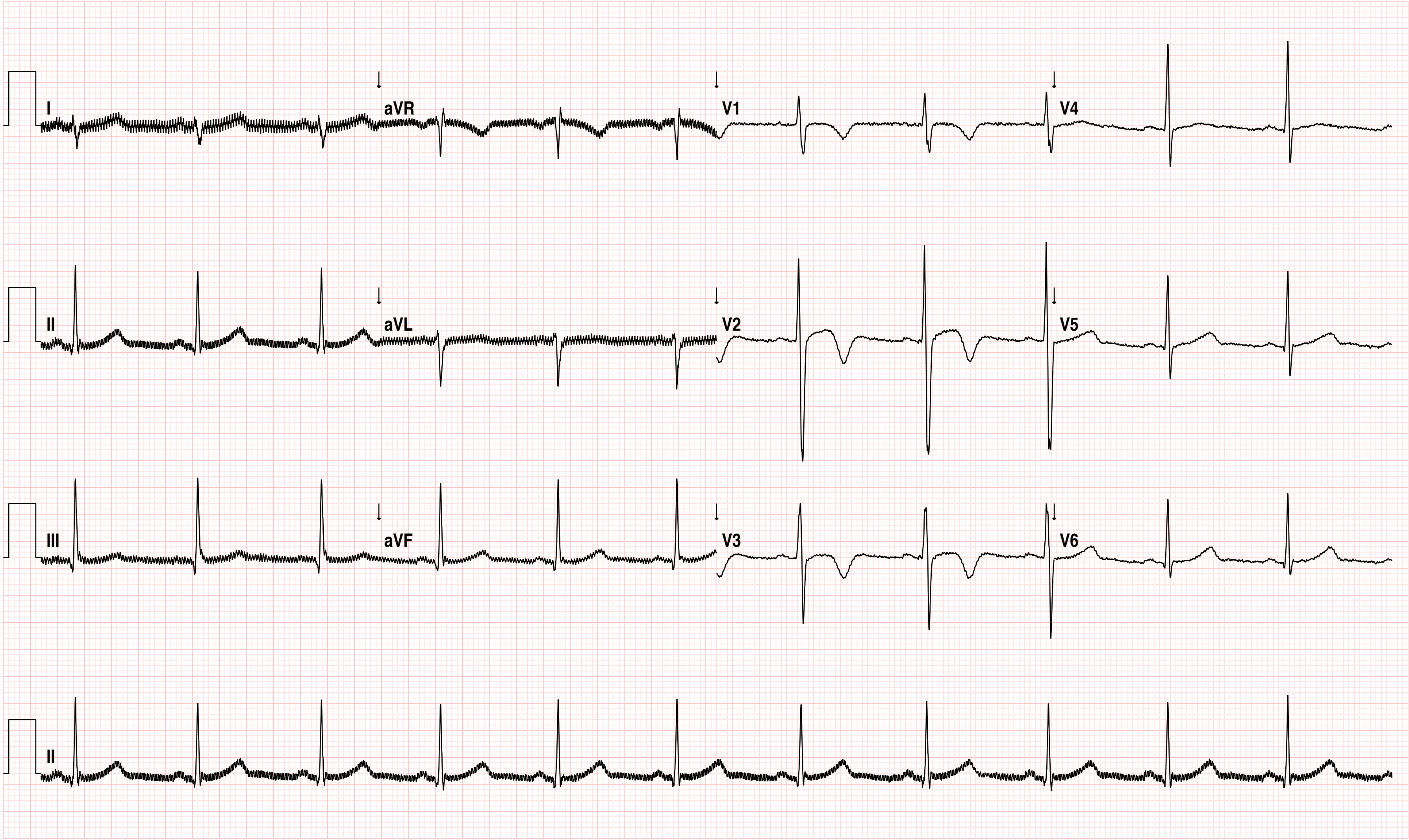

Site # 0 Cart # 0 ELI Link 4.11.6.0 Sequence # 00110 25mm/s 10mm/mV 0.05-300 Hz W

,
ID:
DOB: 24-Nov-1983
34yr, Female

Vent rate: 47 BPM
PR int: 138 ms
QRS dur: 98 ms
QT/QTc: 488 / 450 ms
P-R-T axes: 64 78 43

Reviewed by ________________

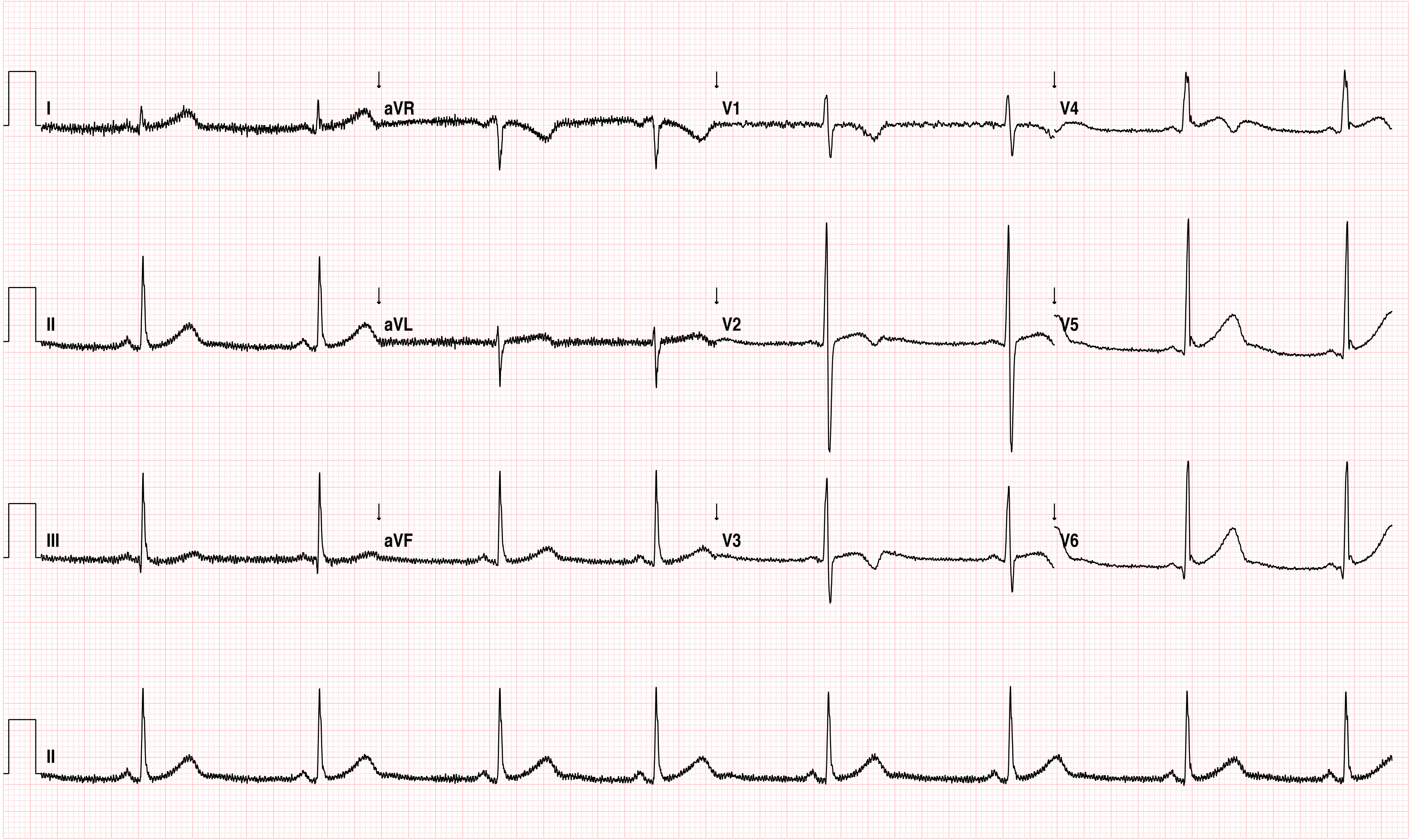

Site # 0 Cart # 0 ELI Link 4.11.6.0 Sequence # 00123 25mm/s 10mm/mV 0.05-300 Hz W

,
ID:
DOB: 13-Mar-1986
32yr, Female

Vent rate: 47 BPM
PR int: 133 ms
QRS dur: 105 ms
QT/QTc: 474 / 439 ms
P-R-T axes: 33 67 44

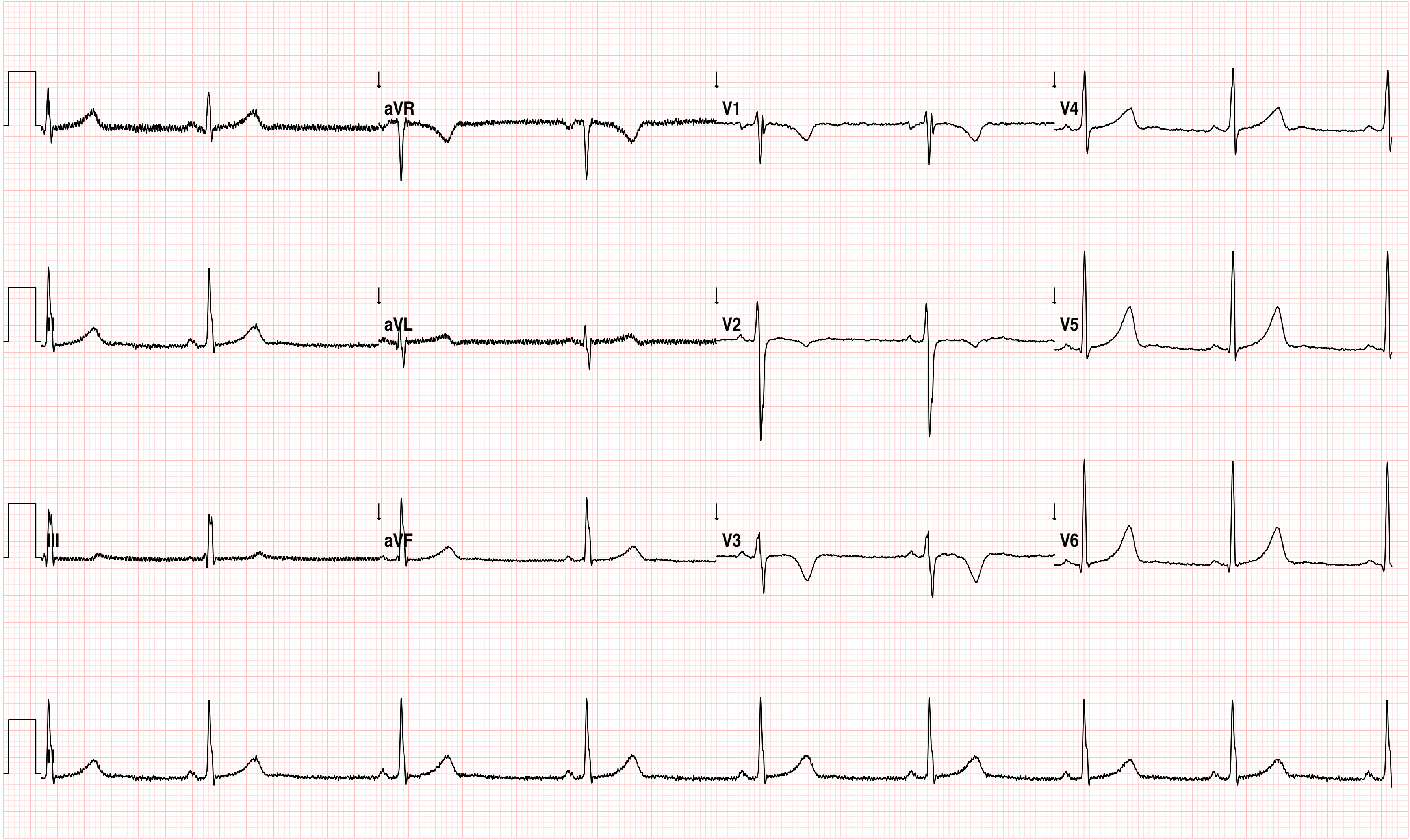

Site # 0 Cart # 0 ELI Link 4.11.6.0 Sequence # 00140 25mm/s 10mm/mV 0.05-300 Hz W

,
ID:
DOB: 20-May-1985
33yr, Female

Vent rate: 50 BPM
PR int: 183 ms
QRS dur: 94 ms
QT/QTc: 535 / 508 ms
P-R-T axes: 13 77 42

Reviewed by ________________

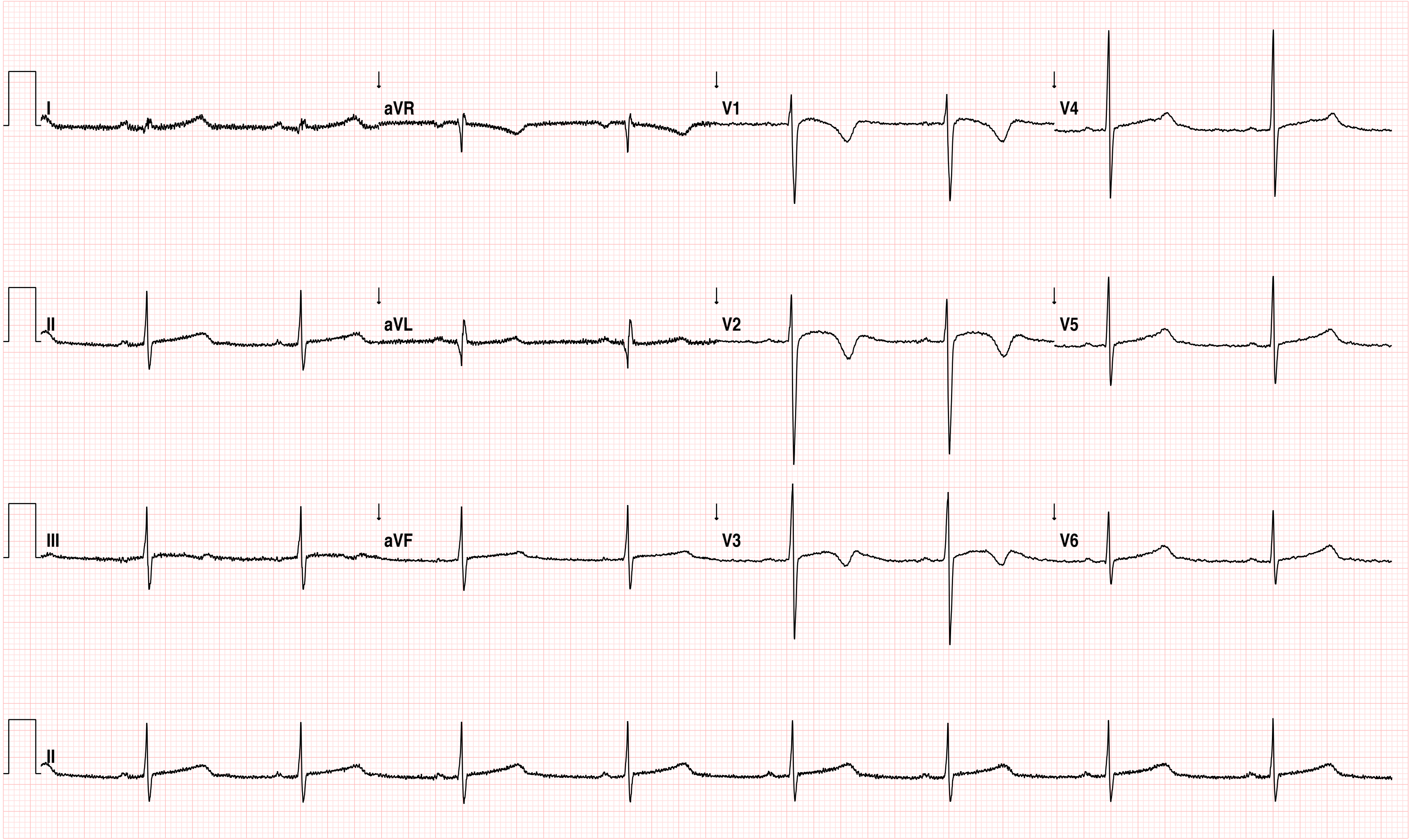

Site # 0 Cart # 0 ELI Link 4.11.6.0 Sequence # 00174 25mm/s 10mm/mV 0.05-300 Hz W

,
ID:
DOB: 23-Sep-1979
38yr, Male

:

Vent rate: 47 BPM
PR int: 146 ms
QRS dur: 92 ms
QT/QTc: 465 / 427 ms
P-R-T axes: 59 84 28

Reviewed by ________________

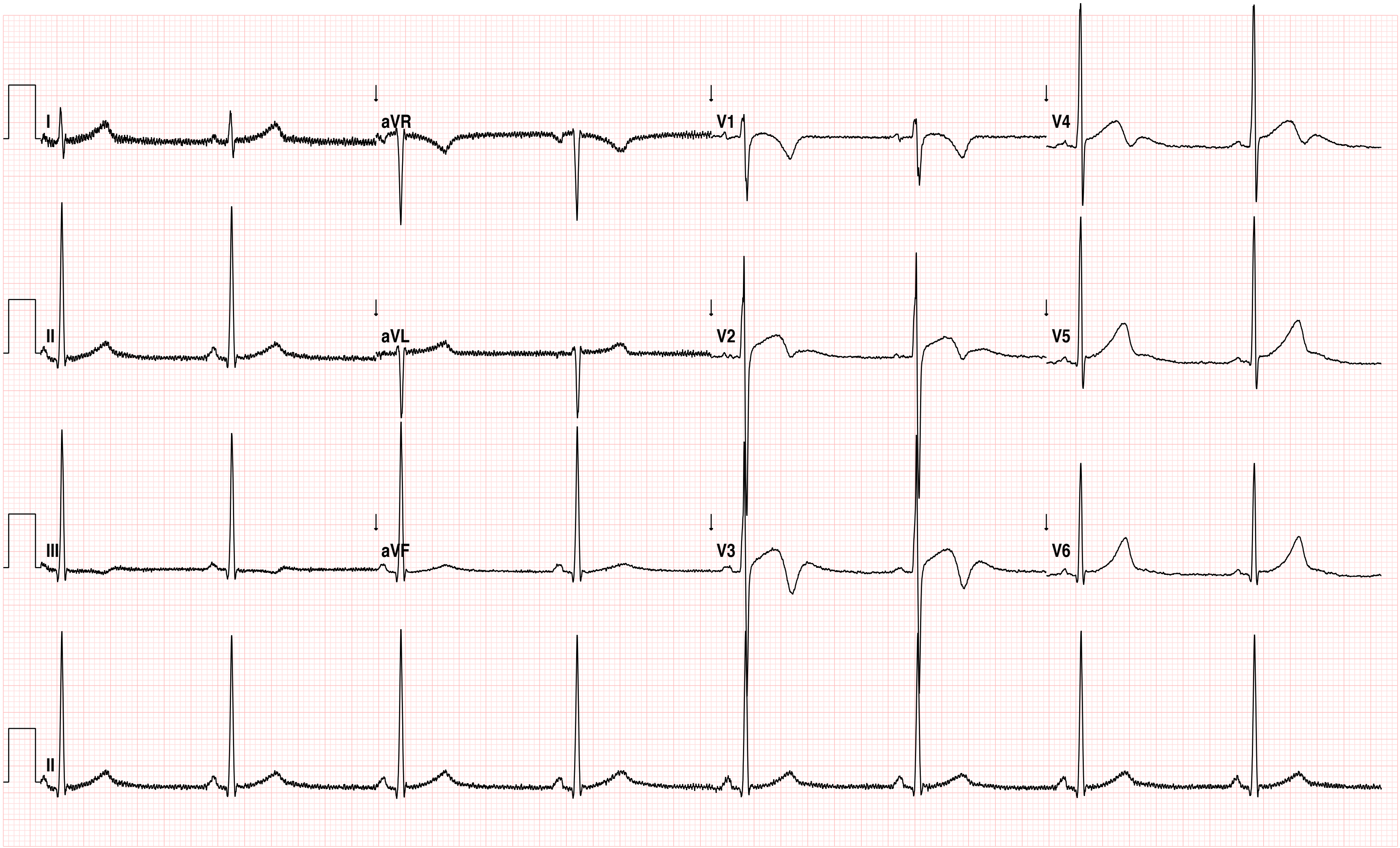

Site # 0 Cart # 0 ELI Link 4.11.6.0 Sequence # 00187 25mm/s 10mm/mV 0.05-300 Hz W

,
ID:
DOB: 06-Dec-1991
26yr, Female

Vent rate: 53 BPM
PR int: 139 ms
QRS dur: 85 ms
QT/QTc: 431 / 413 ms
P-R-T axes: 66 73 43

Reviewed by ________________

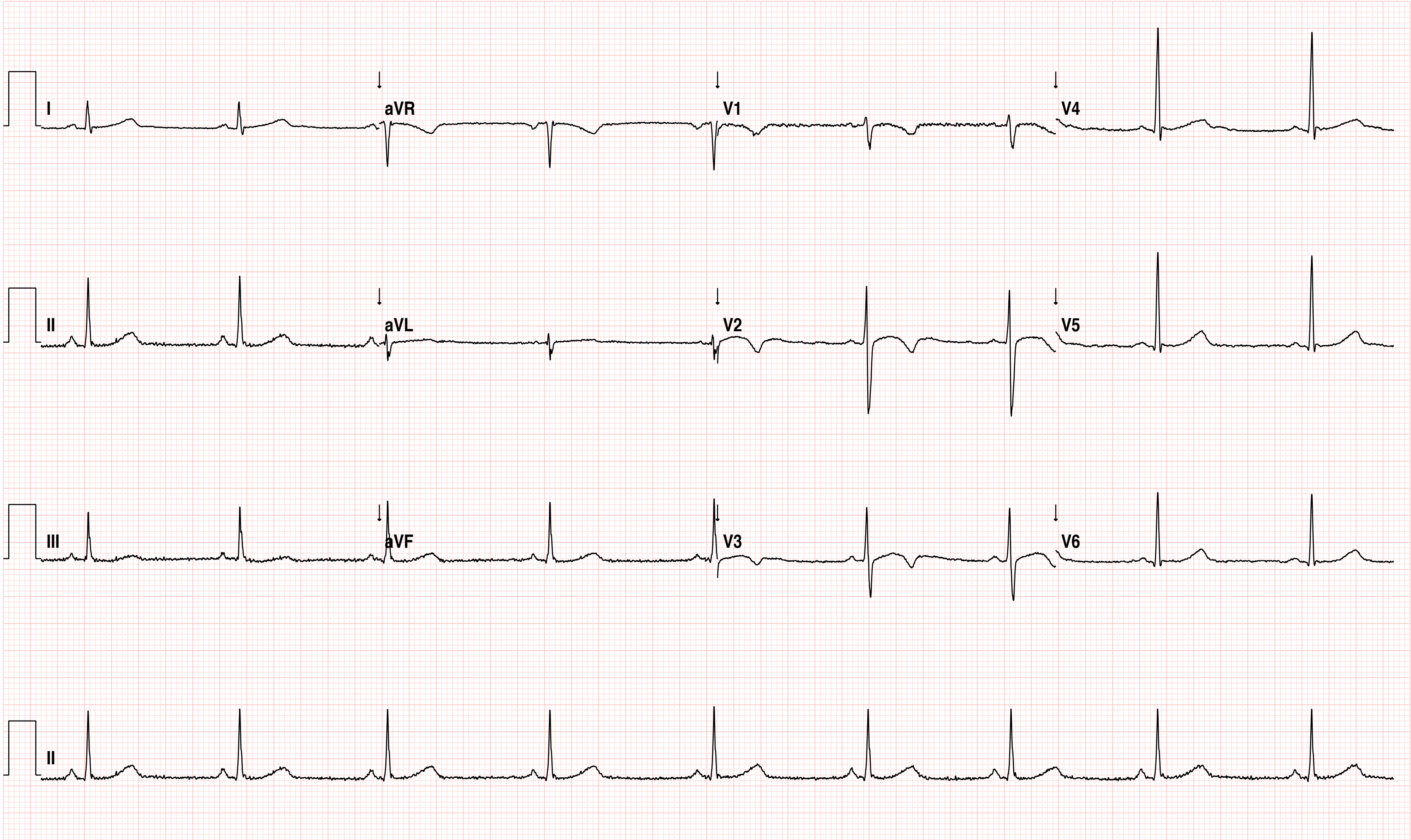

Site # 0 Cart # 0 ELI Link 4.11.6.0 Sequence # 00298 25mm/s 10mm/mV 0.05-300 Hz W
